# QiShenYiQi pill activates autophagy to attenuate reactive myocardial fibrosis via the PI3K/AKT/mTOR pathway

**DOI:** 10.18632/aging.202482

**Published:** 2021-02-11

**Authors:** Shichao Lv, Peng Yuan, Chunmiao Lu, Jianping Dong, Meng Li, Fan Qu, Yaping Zhu, Junping Zhang

**Affiliations:** 1 First Teaching Hospital of Tianjin University of Traditional Chinese Medicine, Tianjin 300193, China; 2 Tianjin Key Laboratory of Traditional Research of TCM Prescription and Syndrome, Tianjin 300193, China; 3 Jiashan Hospital of Traditional Chinese Medicine, Jiaxing 314100, Zhejiang, China; 4 Health Center of Balitai Town, Jinnan, Tianjin 300350, China

**Keywords:** reactive myocardial fibrosis, traditional Chinese medicine, autophagy, PI3K/AKT/mTOR pathway

## Abstract

QiShenYiQi pill (QSYQ), a traditional Chinese medicine, is used to treat cardiovascular diseases. However, the dose-effect relationship of its intervention in the reactive myocardial fibrosis is elusive. In this work, rat models of reactive myocardial fibrosis induced by partial abdominal aortic coarctation were constructed and randomly classified into the model group, 3-methyladenine group, rapamycin group, QSYQ low-dose group, QSYQ medium-dose group, QSYQ high-dose group, and sham-operated rats (control group). We revealed that QSYQ lowered the heart mass index (HMI), left ventricular mass index (LVMI), and myocardial collagen volume fraction (CVF) levels in a dose-dependent mechanism. Additionally, QSYQ increased the number of autophagosomes, and the expression of myocardial Beclin-1 and LC3B. In contrast, it reduced the expression of myocardial p62 and decreased the ratios of myocardial p-PI3K/PI3K, p-Akt/Akt, and p-mTOR/mTOR. In conclusion, our results have revealed that QSYQ impacts anti-reactive myocardial fibrosis in a dose-dependent mechanism which is mediated by the activation of myocardial autophagy via the PI3K/AKT/mTOR pathway.

## INTRODUCTION

Myocardial fibrosis can occur either as reactive fibrosis or reparative fibrosis [1]. Reactive fibrosis, which is often associated with hypertension, is a cardiac hypertrophy and fibrosis reaction to withstand pressure overload and is the primary pathological basis of left ventricular hypertrophy [2]. Notably, left ventricular hypertrophy is a common complication of hypertension. Early compensatory performance is concentric hypertrophy, whereas late decompensation is centrifugal hypertrophy, this causes a gradual decline of cardiac function which consequently leads to heart failure [3]. More than 30% of patients with hypertension may develop left ventricular hypertrophy, and the incidence is positively correlated with the severity of hypertension [4]. Long term pressure overload causes excessive deposition of myocardial collagen fibers. And it significantly elevates collagen concentration, imbalance, and disorder of the proportion and arrangement of various types of collagen, resulting in changes in cardiac function and structure, thus increasing the incidence of cardiovascular events. Hypertension linked with left ventricular hypertrophy can increase the incidence of cardiovascular events such as acute myocardial infarction, congestive heart failure, and sudden death by 6-8 times [5, 6]. Moreover, a meta-analysis of 2449 patients in 5 studies revealed that the absolute risk of cardiovascular events was reduced by 46% in patients with hypertension who had reversed / sustained normal left ventricular hypertrophy [7].

Abnormal remodeling of cardiac tissue characterized by myocardial fibrosis is the core pathological change in various chronic cardiovascular diseases. It is primarily attributed to its long-term delay, recurrent attacks, and long-term treatment. Current medical research has been geared towards exploring multiple drugs that can effectively be utilized to treat myocardial fibrosis. The presently available modern medical treatment of myocardial fibrosis mainly focuses on the renin-angiotensin-aldosterone system (RAAS), among which angiotensin-converting enzyme inhibitors (ACEI), angiotensin receptor inhibitors, and aldosterone antagonists have been extensively studied, and has shown relatively positive results. However, modern medicine only intervenes in a single pathological link. Traditional Chinese medicine (TCM) is advantageous as a multi-component, multi-channel, and multi-target comprehensive intervention, which has an integrated regulatory effect in the treatment of myocardial fibrosis [8]. Taking myocardial fibrosis as the target, the use of TCM in the treatment of cardiovascular disease aims to improve the cardiac microenvironment, promote steady-state recovery, inhibit or reverse myocardial fibrosis, and advance the threshold of disease treatment [9]. Autophagy is a highly conserved biological process mediated by lysosomes in cells, which provides precursors for cell reconstruction, regeneration and repair by degrading biological macromolecules such as nucleic acids, proteins, and organelles thus initiate cell recycling [10]. Studies have shown that autophagy is closely related to myocardial fibrosis. When the heart experiences pressure overload, the cardiac autophagy activity reduces, this is accompanied by high protein synthesis and aggravated myocardial fibrosis, causing cardiac hypertrophy [11]. Besides, the heart can maintain the energy supply of cardiomyocytes and improve ventricular remodeling by activating autophagy under stress [12]. QiShen YiQi compound is composed of *Radix Astragali (Astragalus membranaceus (Fisch.) Bunge, Huangqi), Radix Salviae Miltiorrhizae (Salvia miltiorrhiza Bunge, Danshen), Radix Notoginseng (Panax pseudoginseng Wall. var. notoginseng (Burkill) Hoo et Tseng, Sanqi) and Lignum Dalbergiae Odoriferae (Dalbergia odorifera, Jiangxiang)*. Its dripping pill formulation (QSYQ) was approved by the China State Food and Drug Administration (CFDA) in 2003 and is used clinically to treat cardiovascular diseases. In this study, we used a rat model of abdominal aortic constriction to induce reactive myocardial fibrosis, assess the dose-effect relationship of QSYQ intervention in reactive myocardial fibrosis, and explore its possible mechanism of action from autophagy perspective.

## RESULTS

### Effect of QSYQ on the general morphology of rat heart

**Figure 1:**
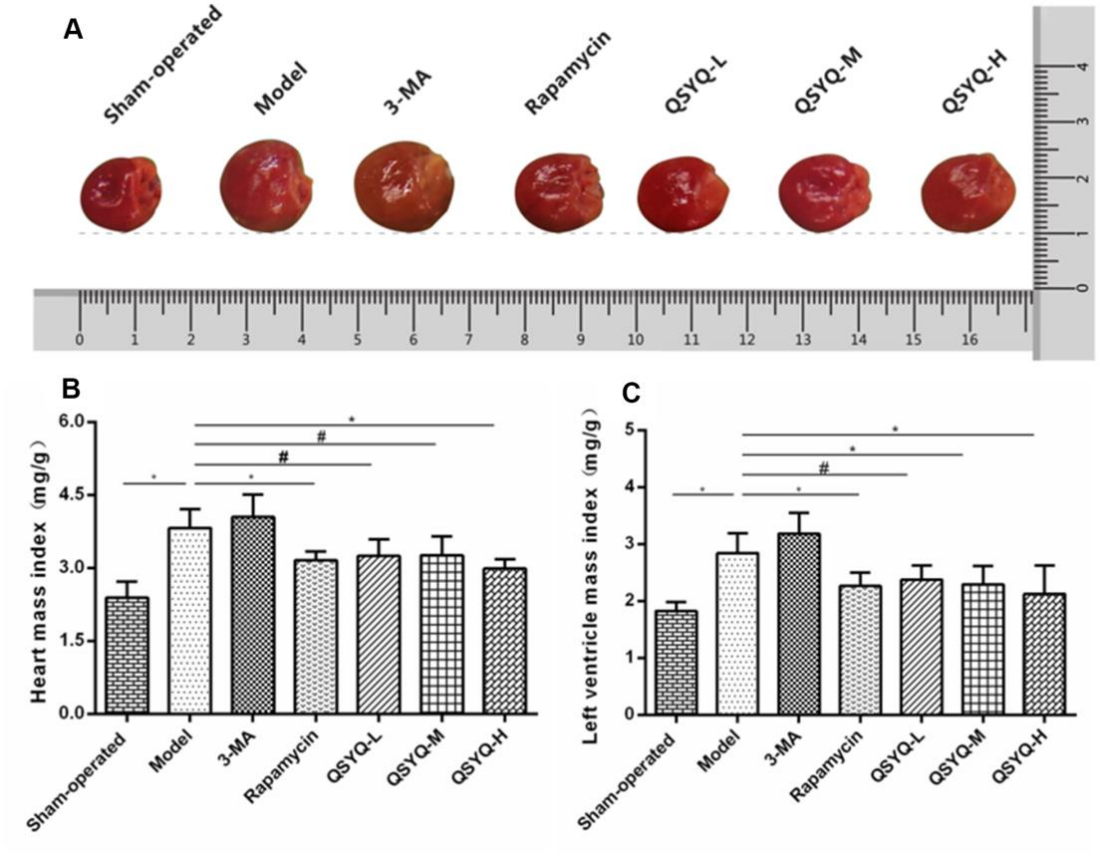
**Effect of QiShen YiQi pill (QSYQ) on the general morphology of rat heart.** (**A**) General morphology of rat heart. (**B**) Heart mass index of rats. (**C**) Left ventricular mass index of rats. Data are expressed as mean ± SD. ^*^P<0.01, ^#^*P*<0.05.

Rats in the model group exhibited significantly larger and high HMI and LVMI in terms of heart size than those in the sham-operated group (*P* < 0.01). The heart of rats in the 3-methyladenine (3-MA) group was enlarged, but the higher HMI and LVMI were not statistically significant compared to the model group (*P* > 0.05). Rats in the rapamycin group exhibited a reduced size of the heart and low HMI and LVMI (*P* < 0.01). However, the HMI and LVMI were reduced in the QSYQ group (*P* < 0.05 or *P* < 0.01). Besides, the high-dose QSYQ group showed significantly lower HMI and LVMI (Figure 1).

### Effects of QSYQ on myocardial histology in rats

**Figure 2:**
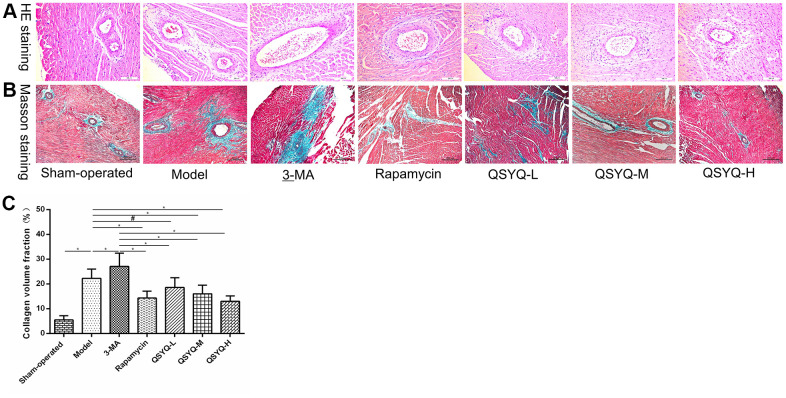
**Effect of QiShen YiQi pill (QSYQ) on myocardial histology in rats.** (**A**) Representative photomicrograph of hematoxylin and eosin (H&E) staining of the myocardium (×200). (**B**) Representative photomicrograph of mason trichrome staining of the myocardium (×200). (**C**) The myocardial collagen volume fraction for each group. Data are expressed as mean ± SD. ^*^P<0.01, ^#^*P*<0.05.

The morphology of the myocardium was normal in the sham-operated group. However, a few collagen fibers were present around myocardial blood vessels, but no prominent collagen fibers existed in the interstitium. The model group showed hypertrophy and swelling of myocardial cells, hypertrophy, and hyperplasia of adjacent fibrous tissues, significant deposition of collagen fibers around myocardial blood vessels and interstitium, and significantly increased collagen volume fraction compared with the sham-operated group (*P* < 0.01). Compared with the model group, those mentioned above myocardial pathological changes were aggravated in the 3-MA group, and the volume fraction of myocardial collagen increased *(P* < 0.01). Besides, the above myocardial pathological changes were reduced, and the volume fraction of myocardial collagen was decreased in the rapamycin and QSYQ groups (*P* < 0.05 or *P* < 0.01). Moreover, the high dose of QSYQ has a tendency to further reduce the myocardial collagen volume fraction (Figure 2).

### Effects of QSYQ on heart structure in rats

**Figure 3:**
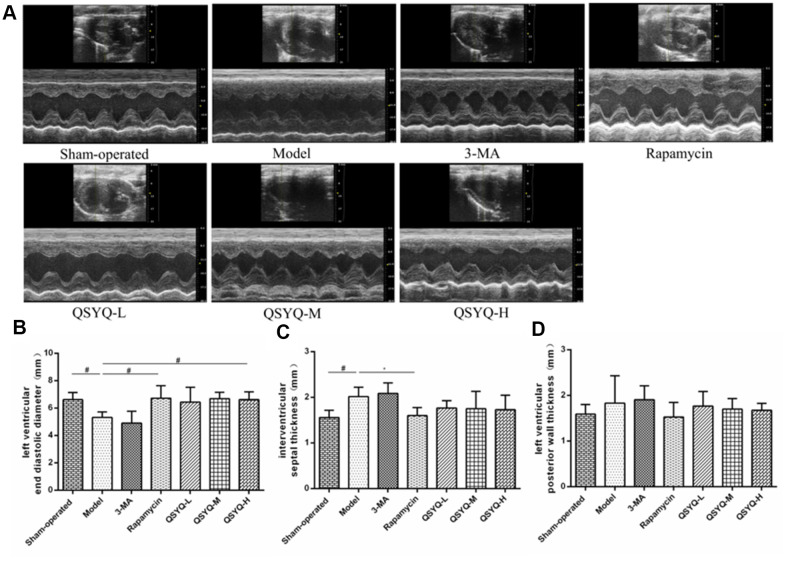
**Effect of QiShen YiQi pill (QSYQ) on heart structure and function in rats.** (**A**) Echocardiography of rat heart. (**B**) Left ventricular end-diastolic diameter in rats. (**C**) Interventricular septal thickness in rats. (**D**) Left ventricular posterior wall thickness in rats. Data are expressed as mean ± SD. ^*^P<0.01, ^#^*P*<0.05.

Compared with the sham-operated group, the left ventricular end-diastolic diameter (LVEDD) was smaller, interventricular septal thickness (IVST) was larger (*P* < 0.05), and left ventricular posterior wall thickness (LVPWT) was not statistically significant in the model group (*P* > 0.05). However, compared with the model group, the LVEDD of the rats smaller decreased, IVST and LVPWT larger in the 3-MA group but the differences were not statistically significant (*P* > 0.05). Rapamycin group showed markedly larger LVEDD (*P* < 0.05), and reduced IVST (*P* < 0.01) and LVPWT (*P* > 0.05). In the QSYQ group, LVEDD was larger, IVST and LVPWT were smaller, notably, the increase in LVEDD was statistically significant (*P* < 0.05), suggesting that QSYQ potentially improved left ventricular hypertrophy in rats (Figure 3). However, the intervention time of QSYQ was short, IVST and LVPWT were not improved significantly, but showed a decreasing trend. In the future study, we will explore the time effect relationship of QSYQ on reactive myocardial fibrosis.

### Effects of QSYQ on myocardial autophagy in rats

**Figure 4:**
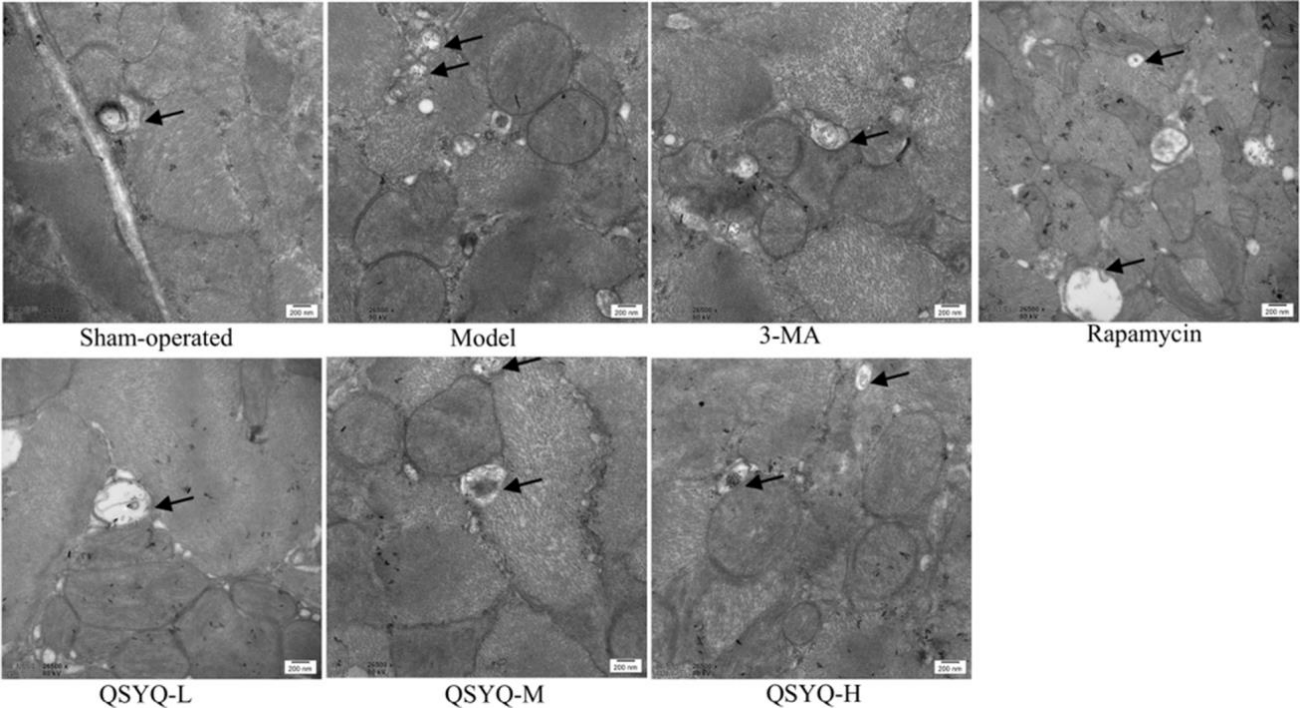
**Effect of QiShen YiQi pill (QSYQ) on myocardial autophagy in rats.** Transmission electron micrographs of rat myocardium in each group, the black arrow show autophagosomes, with a scale of 200nm.

We observed no noticeable pathological changes in the myocardial tissue of the rats. The myocardial fibers were arranged orderly and tightly, with visible horizontal stripes, abundant and visible mitochondria, and a few scattered autophagosomes were visible in the sham-operated group. In the model group, the myocardial fibers were highly disordered in the myocardial cells, with irregular or broken arrangement, blurred horizontal stripes, swollen mitochondria, blurred spine, and an increased number of autophagosomes. Furthermore, the myocardial fiber arrangement was disordered, the mitochondria were swollen with broken ridges, and the number of autophagosomes was lower in the 3-MA group compared to the model group. Besides, in the rapamycin group, the myocardial arrangement of rats was fair, with irregularly shaped mitochondria, and more vacuolated mitochondria and autophagosomes were observed. On the contrary, the intervention of the QSYQ group showed significantly reduced pathological changes in rat myocardial tissue. Also, the myocardial fibers were orderly and closely arranged, mitochondria were abundant, some mitochondrial ridges were swollen and dissolved, and there were more cardiac autophagosomes (Figure 4).

### Effects of QSYQ on beclin-1, LC3B and p62 expression

**Figure 5:**
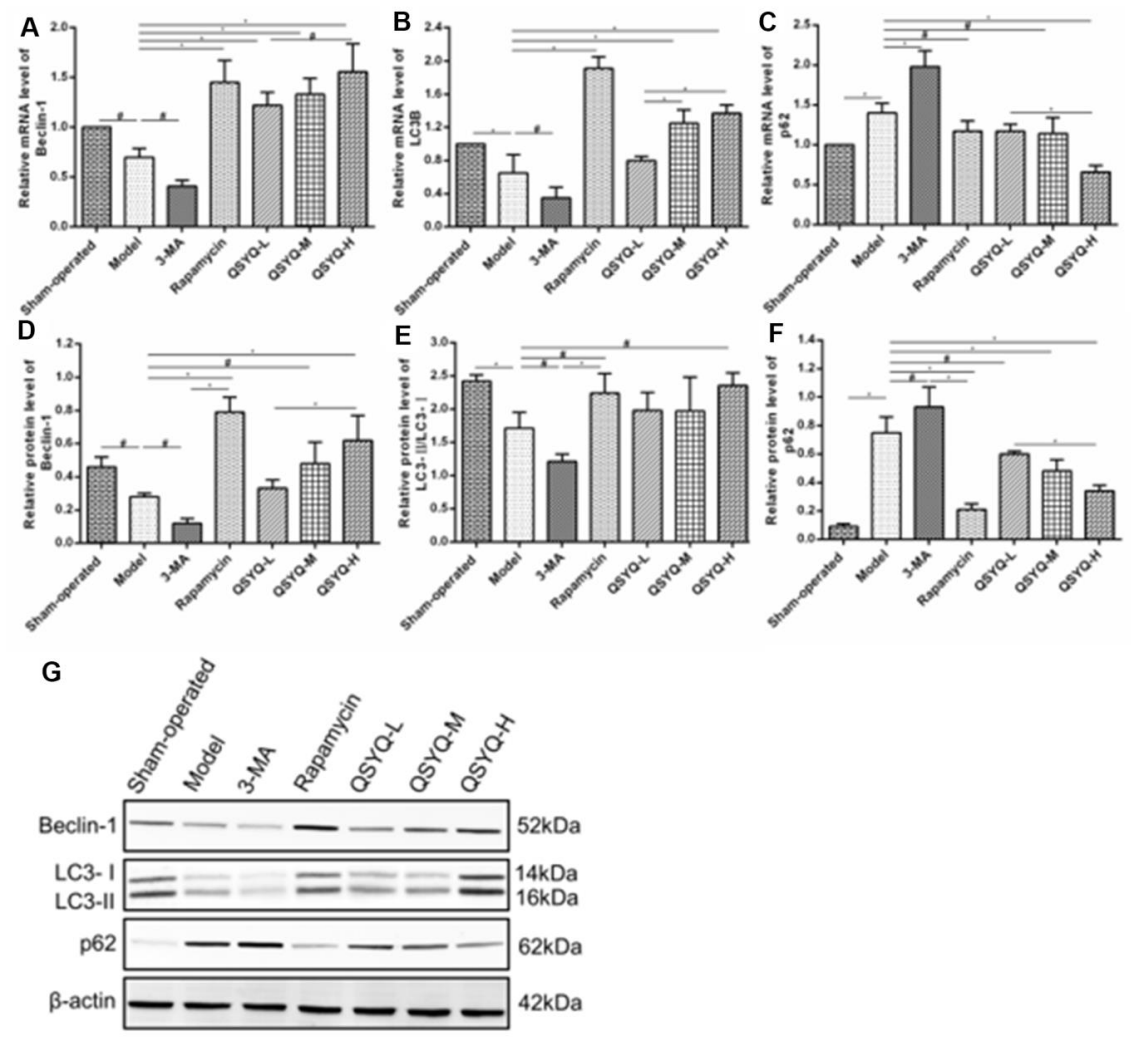
**Effect of QiShen YiQi Pill (QSYQ) on expression of Beclin-1, LC3B, and p62 in rats.** (**A**) The Beclin-1 mRNA expression for each group. (**B**) The LC3B mRNA expression for each group. (**C**) The p62 mRNA expression for each group. (**D**) The relative protein level of Beclin-1 in the myocardium. (**E**) The relative protein level of LC3-II/LC3-II in the myocardium. (**F**) The relative protein level of p62 in the myocardium. (**G**) Beclin-1, LC3-II, LC3-I, and p62 expression in the myocardium of rats in each group as detected by western blot. Data are expressed as mean ± SD, ^*^P<0.01, ^#^*P*<0.05.

Compared with the sham-operated group, the mRNA expression of Beclin-1 and LC3B were significantly lower (*P* < 0.01 or *P* < 0.05), whereas that of p62 was found to be substantially elevated in the model group (*P* < 0.05). Similarly, similar gene expressions pattern was observed in rat cardiomyocytes treated with autophagy inhibitor 3-methyladenine (3-MA). However, rapamycin and QSYQ treatment significantly elevated the expression of Beclin-1, LC3B mRNA *P* < 0.01) while it lowered the expression of p62 mRNA (*P* < 0.01 or *P* < 0.05) (Figure 5).

Western blot results demonstrated that the model group exhibited lower protein expression of Beclin-1, LC3-II/LC3-I (*P* < 0.01 or *P* < 0.05), and higher p62 expression (*P* < 0.01) compared to the sham-operated group. Similar results were observed with 3-MA treatment. Contrarily, the QSYQ compound significantly enhanced the expression of Beclin-1, LC3-II/LC3-I and inhibited the expression of p62. Notably, rapamycin function exerted similar effects (Figure 5).

### Effects of QSYQ on PI3K/AKT/mTOR pathway

**Figure 6:**
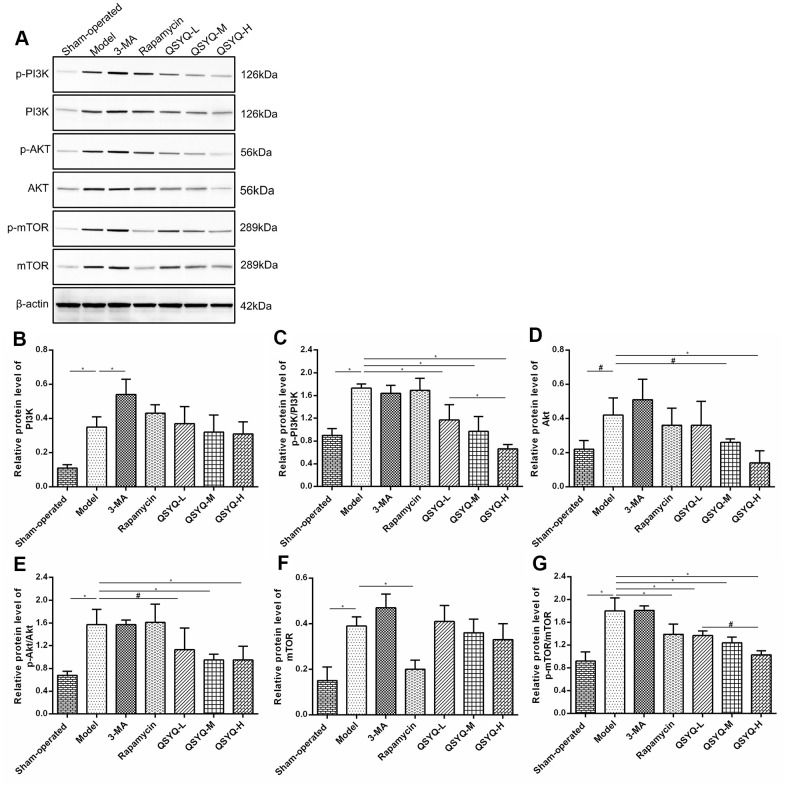
**Effect of QiShen YiQi Pill (QSYQ) on the primary molecules of PI3K/AKT/mTOR pathway.** (**A**) Western blot analysis of protein in the myocardium of rats in each group. (**B**) The relative protein level of PI3K in the myocardium. (**C**) The relative protein level of p-PI3K/PI3K in the myocardium. (**D**) The relative protein level of AKT in the myocardium. (**E**) The relative protein level of p-AKT/AKT in the myocardium. (**F**) The relative protein level of mTOR in the myocardium. (**G**) The relative protein level of p-mTOR/mTOR in the myocardium. Data are expressed as mean ± SD. ^*^P<0.01, ^#^*P*<0.05.

The myocardial PI3K protein, Akt protein, mTOR protein, p-PI3K/PI3K, p-Akt/Akt, and p-mTOR/mTOR in the model group was notably higher than in the sham-operated group (*P* < 0.01 or *P* < 0.05). The differences in myocardial Akt protein, mTOR protein, p-PI3K/PI3K, p-Akt/Akt, and p-mTOR/mTOR in the 3-methyladenine group were not statistically significant (*P* > 0.05) compared to the model group, but PI3K protein expression was significantly increased (*P* <0.01). In contrast, the mTOR protein and the ratio of p-mTOR/mTOR decreased in the rapamycin group (*P* < 0.01), the myocardial PI3K protein, Akt protein, p-PI3K/PI3K, and p-Akt/Akt did not differ statistically (*P* > 0.05). Contrarily, in the QSYQ group, the myocardial Akt protein, p-PI3K/PI3K, p-Akt/Akt and p-mTOR/mTOR ratios decreased in a dose-dependent mechanism (*P* < 0.01 or *P* < 0.05), implying that QSYQ may effectively activate myocardial autophagy and play a role in myocardial protection by regulating the expression of PI3K/AKT/mTOR pathway-related proteins, but the specific targets need to be further studied (Figure 6).

## DISCUSSION

Myocardial fibrosis is a common pathological change that arises from cardiovascular disease. It is mainly characterized by fibroblast proliferation, excessive collagen synthesis, and extracellular matrix deposition [13, 14]. Reactive fibrosis and repairing fibrosis are the main types of myocardial fibrosis. Notably, the former predominantly occur in patients with hypertension, whereas the latter is common in cases of myocardial infarction and other diseases [14]. However, reactive fibrosis and repairing fibrosis are present at different stages of the same disease. For instance, in the rat model of left ventricular overload resulting from aortic constriction, the compensatory performance of the heart to withstand the pressure load is reactive myocardial fibrosis, myocardial cell hypertrophy, without necrosis in the early stage. However, over time, myocardium appears to be decompensated, and myocardial cells begin to partially develop necrosis and apoptosis, which then is repaired through myocardial fibrosis [15]. Hypertension is a highly prevalent disease, whose early induction of reactive myocardial fibrosis potentially causes compensatory hypertrophy of the myocardium, abnormal accumulation of extracellular matrix, and interstitial fibrosis. Consequently, reduced myocardial compliance, dysfunction of relaxation and contraction occur, causing adverse cardiovascular events such as heart failure or arrhythmia [16, 17]. Therefore, the early and timely intervention of reactive myocardial fibrosis is vital in improving the occurrence and progression of cardiovascular diseases such as hypertensive left ventricular hypertrophy and heart failure. HMI and LVMI can reflect the degree of myocardial hypertrophy. The collagen volume fraction can reflect the collagen content in tissues. The myocardial collagen volume fraction of normal rats is about 3% ~ 5%. When the myocardial collagen volume fraction rises to more than 20%, the myocardial contractile function is weakened [18]. Echocardiography can not only accurately reflect the changes of left ventricular systolic function, but also reflect the changes of left ventricular structure. LVEDD, IVST and LVPWT are the main indexes reflecting the structure of the left ventricle.

*Radix Astragali* used to treat heart failure by improving myocardial energy metabolism, strengthening myocardial contractility and improving cardiac function [19]. *Radix Astragali* can improve myocardial energy metabolism, enhance myocardial contractility and improve cardiac function to treat heart failure [20]. *Radix Notoginseng* can alleviate myocardial ischemia and pathological damage of myocardial cells in myocardial tissue, and improve ventricular remodeling [21]. *Radix Salviae Miltiorrhizae* has antioxidant, anti-inflammatory, anti-apoptotic and cardioprotective effects, and is used to treat cardiovascular diseases, including atherosclerosis, myocardial infarction, myocardial hypertrophy, myocardial ischemia-reperfusion (I / R), and chronic heart failure [22]. *Lignum Dalbergiae Odoriferae* can improve ventricular remodeling, promote angiogenesis, improve myocardial function, and antioxidant stress [23]. Studies have shown that QSYQ can reduce myocardial ischemia/reperfusion (I/R) injury [24, 25], inhibit myocardial fibrosis induced by pressure overload [26–28], delay ventricular remodeling caused by ligation of the anterior descending coronary artery [29–31], and improve myocardial injury induced by Adriamycin [32, 33]. Network pharmacology also showed that QSYQ had multiple compounds, multiple targets and various pathways [34–36]. In this study, we established the rat model of reactive myocardial fibrosis with abdominal aortic coarctation. And we revealed that QSYQ could reduce myocardial collagen volume fraction, HMI and LVMI, increase LVEDD. This suggested that QSYQ exerts an anti-reactive myocardial fibrosis effect in a dose-dependent mechanism. Notably, the impact of high-dose QSYQ on anti-reactive myocardial fibrosis was highly significant.

The mechanism of myocardial fibrosis is complex and is yet to be fully elucidated. In recent years, studies have found that myocardial fibroblast activation, inflammatory response, transformed growth actor-β, renin-angiotensin-aldosterone system (RAAS) activation, and autophagy are closely associated with the occurrence and progression of myocardial fibrosis [37–42]. Transmission electron microscopy is the most reliable and classical method to assess autophagy, and the formation of autophagosome is the gold standard in judging autophagy [43, 44]. Beclin-1 participates in the construction of autophagosomes, and its expression level can reflect the occurrence of autophagy [45]. Microtubule-associated protein 1, light chain 3 (LC3) is a marker protein on the autophagosome membrane and is generated in two forms including LC3-I and LC3-II. During autophagy, LC3-I is converted to LC3-II and recruited into an autophagosome, a critical step in the formation of autophagosomes. At this time, P62, as the substrate of LC3-II, forms a complex with it and is eventually degraded by autophagic lysosome [46, 47]. Therefore, Beclin-1, LC3, and p62 are the most critical proteins for autophagy and are highly utilized as autophagy markers. Herein, we revealed that myocardial fibers of the QSYQ group were arranged orderly and tightly with horizontal grain clarity, and rich mitochondria. Moreover, part of the mitochondria appeared to swell and dissolve, and Beclin-1, LC3-II/LC3-I were highly expressed, while expression of p62 was lower compared to the model group. Notably, it was suggested that QSYQ could activate autophagy in rat myocardium in a dose-dependent manner, revealing a highly significant effect of high-dose QSYQ on autophagy.

The PI3K/Akt-mTOR pathway and critical conditions of autophagy can initiate the pathological fibrotic response [48]. Previous studies have shown that inhibition of the PI3K/Akt pathway lowered the expression of PI3K, Akt, I and III collagen in models of diabetes causing cardiomyopathy, thus the progression of cardiac fibrosis is impeded [49]. Three classes of PI3Ks (I, II, and III) have been described, among which type I is widely studied. When cells experience external stress, activated PI3K-I catalyzes the phosphorylation of phosphatidylinositol 2 (PIP2) into the second messenger phosphatidylinositol 3 (PIP3). PIP3 binds to the intracellular signal proteins Akt and phosphoinositide-dependent protein kinase-1 (PDK1) via the PH domain, promoting phosphorylation and activation of Akt [50]. mTOR function both as a negative regulator of autophagy by inhibiting the generation of ULK complex and blocking autophagosome formation, and is a downstream target of the PI3K/Akt pathway. Activated Akt can either directly act on mTORC1 or inhibit the formation of the TSC-1/2 complex, and this activates mTOR causing autophagy [51, 52]. In the mouse model of left ventricular hypertrophy induced by coarctation of the aorta, inhibiting PI3K/Akt-mTOR expression could prevent the development of left ventricular hypertrophy and cardiac insufficiency [53]. In this study, compared with the model group, QSYQ could effectively activate autophagy and decrease the ratio of p-PI3K/PI3K, p-Akt/Akt, and p-mTOR/mTOR for myocardial protection.

## CONCLUSIONS

QSYQ activates autophagy in rat cardiomyocytes by regulating the PI3K/Akt-mTOR pathway and exert an anti-reactive myocardial fibrosis effect. Notably, this provides the basis for treating hypertensive left ventricular hypertrophy and other cardiovascular diseases.

## MATERIALS AND METHODS

### Animals

Male Wistar rats in SPF grade weighing between 220-250g were purchased from Beijing Vital River Laboratory Animal Technology Co., Ltd. [Certificate No.: SCXK (Beijing) 2016-0011]. The rats were maintained on a 12-hour circadian rhythm and allowed to consume water freely, in an environment with a temperature of 22±2° C and relative humidity of 55%±10%. This protocol was conducted in strict compliance with the Guide for the Care and Use of Laboratory Animals (NIH Publication No.85-23, revised in 1996), published by the US National Institute of Health. It was approved by the Animal Ethics Committee of Tianjin University of Traditional Chinese Medicine Approved (No.TCM-LAEC2016016).

### Main reagents

The key reagents used for the experiments were purchased as follows: QiShenYiQi Pills (Tasly Pharmaceutical Group Co. Ltd.), 3-methyladenine (ApexBio Technology, A8353), Rapamycin (LC Laboratories, R-5000), Hematoxylin-Eosin Staining Kit (Beijing Leagene Biotechnology Co., Ltd., DH0006), Masson’s Trichrome Staining Kit (Beijing Leagene Biotechnology Co., Ltd., DC0032), RIPA Lysis Buffer (Beijing Leagene Biotechnology Co., Ltd., PS0012), Phosphatase Inhibitor (Beijing Leagene Biotechnology Co., Ltd., PI0075), Protease Inhibitor Mixture (Beijing Leagene Biotechnology Co., Ltd., PI0015), BCA Protein Quantification Kit (Wuhan Boster Biological Technology Co., Ltd., AR0146), ECL Chemiluminescence Kit (Proteintech Group, Inc, USA, B500022), p-PI3K Antibody (Abcam, ab182651), PI3K Antibody (Proteintech Group, Inc, USA, 20584-1-AP), p-Akt Antibody (Cell Signaling Technology, Inc, USA, 4060), Akt Antibody (Proteintech Group, Inc, USA, 10176-2-AP), p-mTOR Antibody (Cell Signaling Technology, Inc, USA, 2971), mTOR Antibody (Abcam, ab2732), LC3B Antibody (Proteintech Group, Inc, USA, 18725-1-AP), Beclin-1 Antibody (Proteintech Group, Inc, USA, 11306-1-AP), p62 Antibody (Proteintech Group, Inc, USA, 18420-1-AP), β -actin Antibody (Proteintech Group, Inc, USA, 66009-1-Ig), Horseradish Peroxidase AffiniPure Goat Anti-House IgM (H+L) (Proteintech Group, Inc, USA, SA00001-1), Horseradish Peroxidase AffiniPure Goat Anti-Rabbit IgG (H+L) (Proteintech Group, Inc, USA, SA00001-2), RNA Extraction Kit (Takara Biomedical Technology (Beijing) Co., Ltd., 9108), TransScript First-Strand cDNA Synthesis SuperMix (Beijing Transgen Biotechnology Co., Ltd., AT-301-03), PowerUp™ S YBR™ Green Master Mix (Thermo Fisher Scientific, Inc, USA, A25742).

### Reactive myocardial fibrosis rat model

We constructed a rat model of reactive myocardial fibrosis using abdominal aortic coarctation [54]. Groups of rats were fed for 3-5 days. Before surgery, rats were left to fast for 12 h but with free drinking water. We obtained the weight of the rats, after which they were anesthetized with 3% pentobarbital sodium (45mg/kg) via intraperitoneal injection. Disappearing corneal reflex and low muscle strength signified that the anesthesia was completed, rats were then positioned on the operating table. In the middle abdominal region, the rats were shaved at a size of 3*5cm^2^ and disinfected with iodophor. A 2.0-3.0 cm vertical incision was made below 0.5cm on the lower edge of the left costal arch and 0.5cm to the left of the midline of the abdomen to open the abdominal cavity in layers. To fully expose the abdominal aortic segment, we pushed the intestine to the right side of the abdominal cavity, while gently pushing the stomach and spleen upwards. The abdominal aorta at 0.5cm on the branch of the right renal artery was isolated, and we placed a No.7 needle (0.7mm in diameter) in a parallel position. A ligature was tightened between the abdominal aorta and the No. 7 needle with No. 4 surgical sutures (about 0.3mm in diameter), we then removed the needle. With the organs reset, 200,000 U penicillin solution was dripped into the abdominal cavity, then the muscles and skin were sutured layer by layer. Significantly, the rats were kept warm enough and given a mixture of sugar and salt (4:1) after the operation. When fully awakened, the rats were caged back, and intramuscularly injected with 200,000 U/d penicillin for 3 days to prevent infection. The procedures of the sham-operated group were performed identically through laparotomy only with the thread hooked up without ligation.

### Animal grouping and drug administration

The mice were randomized into 6 groups: model group (administered the same volume of distilled water by gavage), 3-methyladenine group (15mg/kg by intraperitoneal injection), rapamycin group (2mg/kg by intraperitoneal injection), QSYQ low-dose group (135mg/kg by gavage), QSYQ medium-dose group (270mg/kg by gavage), and QSYQ high-dose group (540mg/kg by gavage). Sham-operated mice were used as a control group (administered the same volume of distilled water by gavage). The dosage of the QSYQ compound was determined based on the body surface area, which was dissolved into a solution with distilled water for gavage [55].

**Figure 7:**
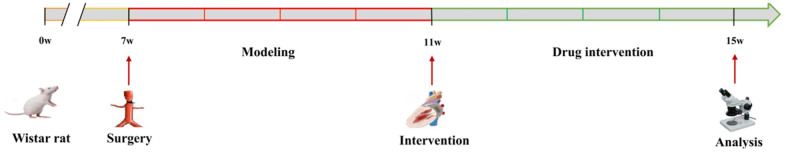
**A flow diagram showing experimental design.**

Blood and tissue specimens were collected after 4 weeks of drug intervention. Firstly, the body mass (BM) of rats was determined. We collected samples under intraperitoneal 3% pentobarbital anesthesia (45mg/kg). Immediately, blood samples were timely collected from the abdominal aorta and centrifuged (3000r/min, 10min), then, serum was collected. Subsequently, the hearts of the rats were removed with their chests open, lavaged with 4° C normal saline, and blotted dry with filter paper. Thereafter, the shape and surface lesions of the heart were assessed and the heart mass (HM) was weighed after the tissues and large blood vessels around the heart were removed. The atria and right ventricular free wall were dissected away before weighing the left ventricle mass (LVM) including the left ventricle and the interventricular septum. After that, according to the data, the heart mass index (HMI) and left ventricular mass index (LVMI) could be respectively calculated as the ratio of HM to body weight (BW) and LVM to BW (HMI=HM/BM (mg/g), LVMI=LWH/BM (mg/g)). Part of the removed heart tissue samples was fixed in 4% neutral formaldehyde solution and quickly snap-frozen in liquid nitrogen. In the end, the serum and heart tissue specimens were immediately transferred to a refrigerator at -80° C for storage (Figure 7).

### Echocardiography to assess cardiac structure and function

Rats were anesthetized through intraperitoneal injection with 3% sodium pentobarbital (45 mg/kg). Then, the rats were fully immobilized in the supine position on an operating platform, and their chest hair was shaved. An ultrasonic coupling agent was used in the chest area to enhance ultrasound transmission. Vevo®2100 ultra-high-resolution ultrasonic imaging system was applied to obtain the left ventricular long axis in two-dimensional patterns. The specific operation was as follows: The sampling line was placed at the maximum left ventricular diameter (papillary muscle level), and the long axis measurement package (PLAX) was measured. Measurement of each index was taken during three cardiac cycles, the mean value was also determined.

### Pathological staining to observe myocardial tissue morphology

Myocardial tissues were fixed in formalin, dehydrated with a conventional ethanol gradient, embedded in paraffin, and cut into 5-μm thick sections. Then, the obtained sections were stained with H&E and Masson. In order to determine the morphological changes of myocardial tissue, we observed the slices under an optical microscope. Notably, 5 visual fields were randomly selected for each section, and the collagen volume fraction was calculated as: (CVF= the area of collagen fiber/area of the total image).

### Transmission electron microscopy of cardiomyocyte autophagosome

Myocardial tissues were fixed in 3% glutaraldehyde and 1% osmium acid, dehydrated with ethanol gradient, embedded in Epon812, cut into semi-thin sections, and stained with toluidine blue. Thereafter the semi-thin sections were localized under light microscopy, cut into ultrathin sections, stained with uranyl acetate and lead citrate. The ultrastructure of cardiomyocytes and autophagosomes was observed under the transmission electron microscope.

### Real-time fluorescence quantitative PCR (RT-qPCR) to detect mRNA expression of target genes in myocardial tissue

Total RNA was extracted from myocardial tissue using the RNAiso Plus reagent. The RNA concentration was assessed using an ultra-fine nucleic acid protein analyzer. Then, RNA was reverse transcribed into cDNA and subjected to RT-PCR using SYBR Green. The specific reaction conditions were as follows: pre-denaturation at 94° C for the 30s, denaturation at 94° C for 5s, annealing, and extension at 60° C for the 30s (40 cycles). Based on the RT-qPCR results, the data were analyzed using the 2^-∆∆Ct^ method. The primers were designed and synthesized by Sangon Biotech (Shanghai) Co., Ltd. The primer sequences were: Beclin-1 upstream primer 5’-TGTTTGGAGATGTTGGAGCA-3’, downstream primer 5’-ATGGAAGGTCGCATTGAAGA-3’; LC3B upstream primer 5’-CGGAGCTTCGAACAAAGAGTG-3’, downstream primer 5’CTTGGTCTTGTCCAGGACGG-3’; p62 upstream primer 5'-GGAGACCCCAAATATGCCC-3', downstream primer 5'-CAGACACCCCACGACCACGAGAGGG-3'. GAPDH was used as an internal reference, its upstream primer was 5’AGATGGTGAAGGTCGGTGTG-3’, while the downstream primer was 5’-CTGGAAGATGGTGATGGGTT-3’.

### Western blot detection of target protein expression in rat myocardium

Here, the myocardial tissue was added to the protein lysis solution, pulverized with an ultrasonic crusher, lysed on ice for 30 min, then centrifuged at 12 000 r/min for 10 min. The supernatant was obtained and the protein concentrations were assessed using the BCA method. Proteins were subjected to SDS-PAGE gel electrophoresis, then electrotransferred to PVDF membranes, blocked in 5% nonfat dry milk for 1h at room temperature. This was followed by an overnight incubation at 4° C with primary antibody [p-PI3K antibody (1:1000 dilution), PI3K antibody (1:1000 dilution), p-Akt (1:2000 dilution), Akt (1:500 dilution), p-mTOR (1:1000 dilution), mTOR (1:2000 dilution), LC3B antibody (1:300 dilution), Beclin-1 antibody (1:1000 dilution), p62 antibody (diluted 1:1000 dilution), β-actin antibody (1:5000 dilution), incubated for 2h at room temperature with the horseradish peroxidase (HRP) labeled secondary antibody(1:5000 dilution)], then stained with ECL reagents, and visualized using an automatic gel imaging system. The Image Lab software was used to evaluate the expression of protein bands. The relative expression of the target protein was calculated using β-actin as an internal reference (Relative expression of target protein = target protein gray value / β-actin gray value).

### Evaluation of myocardial autophagy

Transmission electron microscopy is the gold standard for observing autophagy. The typical structure of autophagy was double-membrane autophagy bodies, which contained residual organelles. Autophagy is a multi-step regulatory process. Beclin-1 is a marker of autophagy activation, and its increased expression can be regarded as a sign of autophagy enhancement. LC3B was a core protein in autophagy formation, and the ratio of LC3-II / LC3-I can estimate the level of autophagy. The level of p62 was closely related to autophagy and was consumed when autophagy occurs. Therefore, autophagy was assessed by analyzing the protein expression of Beclin-1, LC3B, and p62.

### Statistics analysis

Data were presented as mean ± standard deviation (mean ±SD). One-Way Analysis of Variance and LSD test was adopted for data analyses between various groups, *P* < 0.05 denoted statistically significant differences. All statistical data were analyzed using SPSS software.
